# Population aging, macroeconomic changes, and global diabetes prevalence, 1990–2008

**DOI:** 10.1186/s12963-015-0065-x

**Published:** 2015-12-05

**Authors:** Nikkil Sudharsanan, Mohammed K. Ali, Neil K. Mehta, K M Venkat Narayan

**Affiliations:** Population Studies Center, University of Pennsylvania, 239 McNeil Building, 3718 Locust Walk, Philadelphia, PA 19104 USA; Hubert Department of Global Health, Emory University, 1518 Clifton Road NE, Atlanta, GA 30322 USA

**Keywords:** Diabetes, Population aging, Economic growth, Globalization, Urbanization

## Abstract

**Background:**

Diabetes is an important contributor to global morbidity and mortality. The contributions of population aging and macroeconomic changes to the growth in diabetes prevalence over the past 20 years are unclear.

**Methods:**

We used cross-sectional data on age- and sex-specific counts of people with diabetes by country, national population estimates, and country-specific macroeconomic variables for the years 1990, 2000, and 2008. Decomposition analysis was performed to quantify the contribution of population aging to the change in global diabetes prevalence between 1990 and 2008. Next, age-standardization was used to estimate the contribution of age composition to differences in diabetes prevalence between high-income (HIC) and low-to-middle-income countries (LMICs). Finally, we used non-parametric correlation and multivariate first-difference regression estimates to examine the relationship between macroeconomic changes and the change in diabetes prevalence between 1990 and 2008.

**Results:**

Globally, diabetes prevalence grew by two percentage points between 1990 (7.4 %) and 2008 (9.4 %). Population aging was responsible for 19 % of the growth, with 81 % attributable to increases in the age-specific prevalences. In both LMICs and HICs, about half the growth in age-specific prevalences was from increasing levels of diabetes between ages 45–65 (51 % in HICs and 46 % in LMICs). After age-standardization, the difference in the prevalence of diabetes between LMICs and HICs was larger (1.9 % point difference in 1990; 1.5 % point difference in 2008). We found no evidence that macroeconomic changes were associated with the growth in diabetes prevalence.

**Conclusions:**

Population aging explains a minority of the recent growth in global diabetes prevalence. The increase in global diabetes between 1990 and 2008 was primarily due to an increase in the prevalence of diabetes at ages 45–65. We do not find evidence that basic indicators of economic growth, development, globalization, or urbanization were related to rising levels of diabetes between 1990 and 2008.

**Electronic supplementary material:**

The online version of this article (doi:10.1186/s12963-015-0065-x) contains supplementary material, which is available to authorized users.

## Introduction

Diabetes is a rapidly growing contributor to global morbidity and mortality. Studies estimate that 194 million people developed diabetes between 1980 and 2008 and an additional 150–200 million people will develop diabetes by the year 2030 [1–5]. Although diabetes has historically had a higher burden in high-income countries (HICs), the prevalence of diabetes has been rising rapidly in low-to-middle-income countries (LMICs), where 80 % of people with diabetes live [6].

Diabetes prevalence can increase in two ways: (1) as populations age, the incidence and prevalence of diabetes can increase simply due to more people living to older ages, where the risk of diabetes is generally higher compared to younger ages; and (2) the prevalence of diabetes can increase if the age-specific incidence increases over time [7]. Although population aging is recognized as an important cause of increasing diabetes prevalence, the share of diabetes growth over the past 20 years attributable to population aging is unknown [4, 8]. Furthermore, it is unknown how much of the difference in diabetes prevalence across national income groups is due to the older age distribution of HICs compared to LMICs [9].

The contribution of macroeconomic changes to rising levels of diabetes is also unclear—while studies have theoretically linked urbanization and globalization to rising prevalence of non-communicable disease, little empirical evidence exists confirming these relationships [10–12]. Studies of economic growth and life expectancy have found that for some countries increases in life expectancy are correlated with economic growth [13–16], while for others, life expectancy has increased in the absence of economic growth [17, 18]. However, these types of analyses have not been extended to diabetes.

The primary aims of this paper are: (1) to determine the contribution of population aging to the growth in diabetes prevalence between 1990 and 2008; (2) to determine the contribution of age composition to differences in diabetes prevalence between HIC and LMICs; and (3) to identify macroeconomic changes that were associated with the growth in diabetes prevalence.

## Methods

We used published cross-sectional data on age and sex-specific counts of people with diabetes by country, national population counts, and country-specific macroeconomic variables for the years 1990, 2000, and 2008.

### Diabetes and population sizes

Counts of the number of people aged 20–100 with diabetes by 10-year age and sex groups for 193 countries in 1990 and 2008 were obtained from recent data published by Danaei and colleagues [1]. These data combine individuals with both type 1 and type 2 diabetes. To estimate counts of people with diabetes by year-country-sex-age groups, Danaei and colleagues collected national data on multiple glycemic metrics (including but not limited to hemoglobinA1c, mean postprandial glucose, and fasting plasma glucose) from a combination of health examination surveys and epidemiological studies. Regression models were used to standardize and express all glycemic data in terms of fasting plasma glucose (FPG). Diabetes prevalence was then estimated for each year-country-sex-age group using the American Diabetes Association classification of diabetes (FPG of 7 mmol/L or greater). This prevalence was then applied to the population size for each group to calculate the number of people with diabetes. Where there were missing data, Danaei and colleagues used a Bayesian Hierarchical model to predict counts of people with diabetes (the predictive model had strong predictive validity: the 95 % uncertainty intervals for predicted FPG included 95–98 % of the actual values). Additional steps were taken to capture uncertainty and rural-urban differences in diabetes prevalence. Further details of the methodology were presented in Danaei and colleagues [1].

Age-sex-specific population estimates were collected from the United Nations World Population Prospects 2010 report for the years 1990–2008. Counts of population in these data were extracted from a combination of national censuses, official government estimates, and surveys. The United Nations Department of Economic and Social Affairs, Population Division corrected the data for misreporting, missing data, and other error [9].

We define diabetes prevalence as the share of individuals living with diabetes; we estimate diabetes prevalence by dividing the number of individuals with diabetes in a population by the total size of that population.

### Macroeconomic measures

Despite limited research on the relationship between diabetes prevalence and macroeconomic variables, the literature on the relationship between mortality and macroeconomic changes is rich. Drawing from the literature on the relationship between mortality and macroeconomic changes, we identified the following variables as potential correlates of diabetes prevalence: gross domestic product (GDP) per capita, foreign direct investment net inflows, percent of the population residing in urban areas, female labor force participation, and health expenditure per capita.

Estimates of GDP per capita, foreign direct investment net inflows, percent of the population residing in urban areas, female labor force participation, and health expenditure per capita for 159 countries were extracted from the World Bank World Development Indicator database for the years 2000 and 2008 [19]. Data for 1990 were not used since many of the countries were missing macroeconomic information for that year. Gross domestic product per capita, health expenditure per capita, and foreign direct investment were all standardized by purchasing power parity. For more information, see World Bank World Development Indicators, [19]. We dropped the United Arab Emirates, St. Lucia, Equatorial Guinea, and Maldives due to implausible values. Using the World Bank cutoff for national income, countries with a 2012 gross national income per capita of $12,616 or more were classified as high-income countries, while countries that did not meet this level were classified as low-to-middle-income countries.

### Statistical analysis

We began by quantifying the contribution of population aging to the growth in diabetes prevalence between 1990 and 2008 using decomposition approaches [20] Additional file 1. The decomposition identifies how much of the growth in diabetes prevalence can be attributed to populations getting older and how much is due to changes in the age-specific prevalence. The share of diabetes growth attributable to changes in the age-specific prevalence was further decomposed to identify the percent contribution of each age group. We first decomposed the change in diabetes prevalence for the global population, then for HICs and LMICs separately. We next examined the role of age-compositional differences to differences in diabetes prevalence between HICs and LMICs. Within each year (1990 and 2008), we combined age-specific prevalence estimates from LMICs with the age composition of HICs. This approach allowed us to standardize the age distributions of the two national income groups providing an estimate of what the prevalence of diabetes in LMICs would be if they had the age distribution of HICs.

We next estimated the relationship between diabetes prevalence and macroeconomic indicators using bivariate correlations and multivariate ordinary least squares (OLS) regression. Compared to decomposition, these approaches are better suited for estimating multivariate relationships over multiple time points, making these methods relevant for our analysis using many macroeconomic variables measured over time.

Because differences in the age composition of countries could affect our estimates, we began by age-sex standardizing diabetes prevalence to the 2000 world population. We then took the first difference value of all variables by subtracting a country’s responses on each variable in 2008 by their response on that same variable in 2000. Our primary outcome was the change in diabetes prevalence between 2000 and 2008. By modeling the change in diabetes prevalence on the change in the macroeconomic variables, we eliminate the effect of time-invariant differences between countries that may be related to both diabetes prevalence and the macroeconomic variables.

The bivariate relationship between the change in diabetes prevalence and the change in each of the macroeconomic variables was estimated using the Kendall Tau rank correlation coefficient. We plotted the LOWESS estimation curve, which estimates the shape of the bivariate correlations. Finally, we used a multivariate OLS regression to estimate the relationship between diabetes prevalence and all the macroeconomic variables simultaneously.

## Results

Globally, diabetes prevalence grew by two percentage points between 1990 (7.4 %) and 2008 (9.4 %) (Table 1). This suggests that roughly 150 million more individuals are living with diabetes. This growth was not unique to HICs or LMICs: the prevalence of diabetes in HICs grew from 7.1 to 9.8 %, with a similar level of growth for LMICs (7.5–9.3 %).

**Table 1:** Growth in diabetes between 1990 and 2008 attributable to age compositional changes and age-specific prevalence changes, 193 countries, adults ages 25 to 100

Region	1990 prevalence	2008 prevalence	Growth	% change due to age composition	% change due to age- specific prevalence
Both sexes					
World	7.4 %	9.3 %	1.9 %	19.2 %	80.8 %
HICs	7.1 %	9.8 %	2.7 %	28.2 %	71.8 %
LMICs	7.5 %	9.2 %	1.7 %	20.7 %	79.3 %
Males					
World	7.4 %	9.4 %	2.0 %	20.3 %	79.7 %
HICs	7.4 %	11.0 %	3.6 %	24.3 %	75.7 %
LMICs	7.4 %	9.1 %	1.7 %	22.1 %	77.9 %
Females					
World	7.4 %	9.2 %	1.8 %	18.0 %	82.0 %
HICs	6.8 %	8.6 %	1.8 %	37.2 %	62.8 %
LMICs	7.6 %	9.4 %	1.8 %	19.1 %	80.1 %

Notes: Crude prevalence shown. Countries were classified as high income based on World Bank designations. Estimates for percentage change were taken from a decomposition analysis of the 1990 and 2008 data

Data source: Danaei and colleagues, [1]

Between 1990 and 2008 the prevalence of diabetes increased for both men and women. However, the sex pattern in the level of diabetes differed between HICs and LMICs. In HICs, men had higher levels of diabetes prevalence compared to women for both years (7.4 % versus 6.8 % in 1990; 11 % versus 8.6 % in 2008); in contrast, women had higher levels of diabetes prevalence in LMICs (7.6 % versus 7.4 % in 1990; 9.4 % versus 9.1 % in 2008). Furthermore, although the growth in diabetes prevalence over time was similar for most of the income-sex groups, diabetes prevalence grew substantially more for men in HICs (3.6 percentage point growth compared to 1.7–2.0 percentage points for the other groups).

Overall, population aging was responsible for 19.2 % of the growth in diabetes prevalence, with 80.8 % of the growth attributable to increases in the age-specific prevalence (Table 1). Population aging was slightly more important in HICs compared to LMICs (28.2 % versus 20.7 %). The contribution of population aging also remained relatively consistent by sex. Women in HICs are the main exception to this pattern, as population aging was responsible for a much larger share of their growth in diabetes prevalence compared to the other groups (37.2 % compared to 18.0–24.3 % for the other groups).

**Fig. 1:**
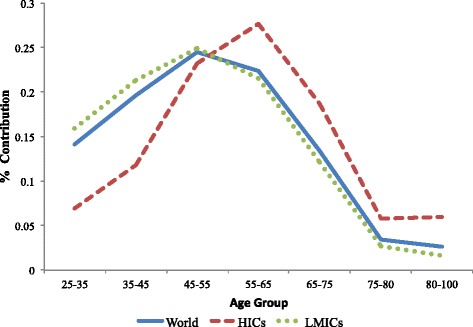
The contribution of each age group to the growth in diabetes prevalence attributable to increasing age-specific prevalences, 1990–2008, 193 countries, adults ages 25 to 100

Figure 1 presents the contribution of each age group to the share of diabetes growth attributable to changes in the age-specific prevalence. In both LMICs and HICs the growth in diabetes between 1990 and 2008 was primarily due to an increase in the prevalence of diabetes between ages 45 and 65. Globally, 24.5 % of the share of diabetes prevalence attributable to changes in the age-specific prevalence was between ages 45 and 55, and 22.4 % between ages 55 and 65. This pattern was similar for LMICs with 24.9 % between ages 45 and 55. In contrast to LMICs, the older middle ages had a larger impact on diabetes growth in HICs (27.7 % attributable to ages 55–65). Importantly, changes in the age-specific prevalence of diabetes at older ages only had a minor impact on the growth in diabetes prevalence between 1990 and 2008 for both LMICs and HICs.

Despite having very different age compositions, HICs and LMICs had fairly similar levels of diabetes prevalence in 1990 and 2008. We observed a small crossover in the prevalence of diabetes between 1990 and 2008: in 1990, LMICs had a slightly higher prevalence of diabetes (7.5 % versus 7.1 %) but by 2008 the prevalence of diabetes was higher in HICs (9.8 % versus 9.3 %). When we estimated what the prevalence of diabetes in LMICs would be if they had the age distribution of HICs (Table 2), we found that LMICs had a higher prevalence of diabetes in both 1990 (9.0 % versus 7.1 %) and 2008 (11.3 % versus 9.8 %).

**Table 2:** Crude and standardized estimates of diabetes prevalence for HICs and LMICs, 193 countries, 1990 and 2008

	1990	2008
Actual		
HICs	7.1 %	9.8 %
LMICs	7.5 %	9.3 %
Difference	0.4 %	−0.5 %
Standardized		
HICs	7.1 %	9.8 %
LMICs	9.0 %	11.3 %
Difference	1.9 %	1.5 %

Notes: For the standardized estimates, LMICs were standardized to the age distribution of HICs for that year

Data source: Danaei and colleagues, [1]

**Fig. 2:**
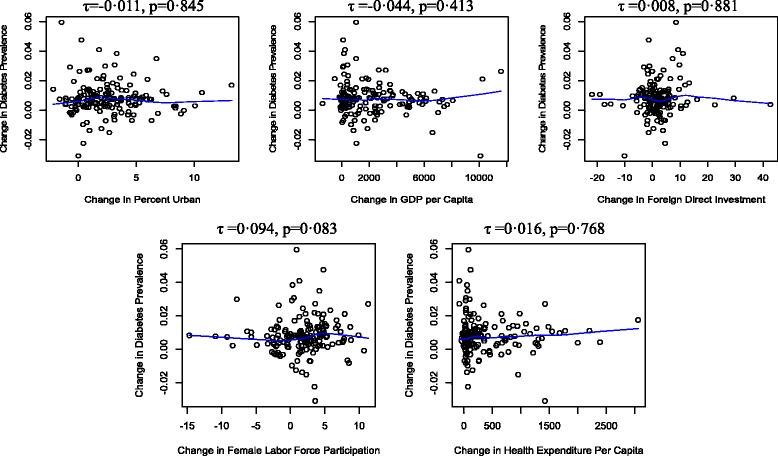
First-difference Kendall Correlation of age-gender-standardized diabetes prevalence and macroeconomic correlates with estimated LOWESS line, 2000–2008, 155 countries. Each point represents one country. τ is the Kendall correlation coefficient; p-values shown test whether τ is statistically different from 0

Figure 2 presents the results of the bivariate analysis. For each scatterplot, we present the correlation coefficient and the corresponding p-value. We did not find evidence that any of the variables were associated with change in diabetes prevalence. Since many of these variables are likely to be correlated with each other, a multivariate model is needed to identify the independent relationship of each variable with the change in diabetes prevalence.

We used a multivariate model to test whether all the macroeconomic variables considered simultaneously were associated with the change in diabetes prevalence, but did not find any independent associations (Table 3). Even when considered jointly, we found no evidence that the macroeconomic variables were associated with the change in diabetes prevalence (F-test *p* = 0.450).

**Table 3:** Estimated multivariate associations between the change in macroeconomic variables and change in age-sex-standardized diabetes prevalence between 2000 and 2008 (*N* = 155)

	Estimated coefficient	Lower 95 % CI	Upper 95 % CI
GDP per capita (*1000)	−0.000742	−0.001682	0.000198
Foreign direct investment (*1000)	0.165000	−0.148000	0.445000
Female labor force participation	0.000163	−0.000306	0.000632
Health expenditure per capita (*1000)	0.001208	−0.003148	0.005564
Percent of the population residing in urban areas	−0.000742	−0.000946	0.000459

F-test *p*-value = 0.450

Notes: Coefficients on GDP per capita, foreign direct investment, and health expenditure per capita represent the change in diabetes prevalence associated with a $1000 increase. Coefficients for female labor force participation and percent of the population residing in urban areas represent the change in diabetes prevalence associated with a 1-percentage point increase

Data source: Danaei and colleagues, [1]; World Bank World Development Indicators, [19]

^*^ *p* <0.05, ** *p* <0.01, *** *p* <0.001

## Discussion

We find that population aging was not the primary cause of increasing global diabetes prevalence over this period. Even when looking at countries by national income, the clear trend is that diabetes prevalence increased as a result of growing age-specific levels of diabetes, specifically at ages 45–65.

Although the prevalence of diabetes looks comparable between HICs and LMICs, differences in demographic composition obscured the underlying levels of diabetes. If LMICs had the age distribution of HICs, the prevalence of diabetes would be 1.5–2.0 percentage points higher in both 1990 and 2008. Given that the percent of the global population above the age of 60 is expected to grow from 10 % in 2000 to 32.2 % in 2100, these results suggest that even if current levels of age-specific diabetes remain constant, the prevalence of diabetes in both HICs and LMICs will increase considerably as their populations age [21].

Consistent with many prior studies, we find that the global prevalence of diabetes increased between 1990 and 2008. However, there were important differences in growth by sex and national income. Men in HICs had consistently higher levels of diabetes compared to women—they also experienced the largest growth in diabetes between 1990 and 2008. In contrast, women had higher levels of diabetes in LMICs.

A large body of work has linked macroeconomic conditions with population health. Using country-specific measurements of GDP per capita to measure economic growth, female labor force participation, and health expenditure per capita as indicators of development, foreign direct investment as an indicator of globalization, and the percent of the population living in urban areas to measure urbanization, we fail to find an association between any of the commonly used macroeconomic measures and diabetes prevalence. Diabetes prevalence may be growing due to rising obesity and shifting dietary patterns that are unrelated to economic growth and development. For example, data from 1962 to 1994 show a weakening relationship between economic growth and fat consumption, potentially because the cost of cheap fats has lowered in countries at all levels of national income [22]. Furthermore, increased economic growth is only empirically associated with increased food consumption at very low levels of GDP, with a pronounced flattening of the relationship at moderate to high levels of income. The composition of food consumption shifts from carbohydrates to fats at higher levels of GDP per capita but this relationship also plateaus, with many notable outliers including Japan [23].

Food cultures may also modify the effect of economic growth on diet and diabetes outcomes [24]. For example, South Korea has retained a largely traditional diet despite rapid economic growth, and has one of the lowest obesity rates in Asia [25]. Further studies elucidating how shifting macroeconomic conditions influence behavioral factors are warranted.

To our knowledge this analysis is one of the first to empirically test the associations between macroeconomic changes and global diabetes prevalence. We were also able to refine estimates of the contribution of population aging by using rich retrospective data, rather than hypothetical projected data that previous studies have relied on. By using longitudinal data with first differences, our analysis controlled for time-invariant differences between countries—providing a robust evaluation of the association between macroeconomic changes and diabetes prevalence.

There are some limitations to our approach. We measured the effect of population aging using 10-year age groups (with the exception of the final age group). For our method of decomposition, 5-year age intervals would have provided a more accurate decomposition result. For groups with missing data, Danaei and colleagues estimated counts of individuals with diabetes based on patterns of diabetes in similar contexts. If the true levels of diabetes in these groups were different from the imputed counts, our estimates of the contribution of population aging and changes in the age-specific levels of diabetes prevalence would be biased. Without knowing the direction of bias for the imputed values, the direction of the bias for our estimates is unclear. Our models do not incorporate Danaei and colleagues’ measures of uncertainty; while incorporating the uncertainty in the predicted counts of individuals with diabetes would provide variance estimates for our main results, it would not change our point estimates of the contributions of population aging and changes in the age-specific levels of diabetes prevalence. Still, since we do not have variance estimates for the estimated contributions, small differences across groups of countries should be interpreted cautiously. Importantly, our macroeconomic indicators are aggregated at the population level and may not capture the complex dimensions of these macroeconomic processes. For example, a single indicator of foreign direct investment is unlikely to capture all aspects of globalization. Other measures of globalization, such as indices of market deregulation, may result in different conclusions [26]. Using individual level data would also provide better insight into the relationship between economic changes and diabetes. We also measured macroeconomic changes over a relatively short time period, 8 years; extending this time range in future work may provide important information on long-run health and economic trends. Finally, the use of partially imputed data could affect the relationship between the macroeconomic variables and diabetes prevalence in two ways. First, the use of imputed data may have affected our coefficient estimates. Since the imputed data are based on prediction models, our estimates of the coefficients may be biased downward if the imputation process for missing groups introduced measurement error into the predicted counts of individuals with diabetes. The coefficients may also be biased if the predicted counts of individuals with diabetes for missing groups are biased. Second, since our regression estimates do not account for the imputation uncertainty, the standard errors for the coefficients on the macroeconomic variables are underestimated. However, given that we do not observe statistically significant relationships, correcting the standard errors to account for imputation uncertainty would not change the significance on any of the coefficients. Further research in this area would benefit from raw diabetes data to determine the extent of the relationship between macroeconomic changes and changes in diabetes prevalence.

## Conclusion

We find that the growth in global diabetes prevalence was not an inevitable side effect of populations growing older. Between 1990 and 2008, the increase in the global prevalence of diabetes was primarily due to an increase in the prevalence of diabetes at ages 45–65. This implies that even if current levels of diabetes across age groups remain constant, the prevalence of diabetes in both HICs and LMICs will continue to rise as their populations continue to age. Diabetes prevalence also appears to be unrelated to multiple indicators of economic growth and appears to be increasing in countries at all levels of national income.

## Additional file

Additional file 1:
**Technical appendix for the decomposition procedures.** (PDF 117 kb)
