# Supplementary material for: Population aging, macroeconomic changes, and global diabetes prevalence, 1990–2008
Source: Popul Health Metr. 2015 Dec 5;13:33. doi: 10.1186/s12963-015-0065-x (PMC4670493; doi:10.1186/s12963-015-0065-x)
Supplement: Additional file 1: — Technical appendix for the decomposition procedures. (PDF 117 kb) [file 12963_2015_65_MOESM1_ESM.pdf]

Population aging, macroeconomic changes, and global diabetes prevalence, 1990-2008

Technical Appendix

1 Definitions

Let:

n^{1990} : population size in 1990

n^{2008} : population size in 2008

n_i^{1990} : population size for age group i in 1990

n_i^{2008} : population size for age group i in 2008

d^{1990} : number of people with diabetes in 1990

d^{2008} : number of people with diabetes in 2008

d_i^{1990} : number of people in age group i with diabetes in 1990

d_i^{2008} : number of people in age group i with diabetes in 2008

The total and age-specific prevalence of diabetes can now be calculated as:

$p^{1990} = \frac{d^{1990}}{n^{1990}}$: prevalence of diabetes in 1990

$p^{2008} = \frac{d^{2008}}{n^{2008}}$: prevalence of diabetes in 2008

$p_i^{1990} = \frac{d_i^{1990}}{n_i^{1990}}$: prevalence of diabetes for age group i in 1990

$p_i^{2008} = \frac{d_i^{2008}}{n_i^{2008}}$: prevalence of diabetes for age group i in 1990

Similarly, the proportionate age distribution can be calculated as:

$c_i^{1990} = \frac{n_i^{1990}}{n^{1990}}$: proportion of the population in age group i in 1990

$c_i^{2008} = \frac{n_i^{2008}}{n^{2008}}$: proportion of the population in age group i in 2008

2 Decomposition of diabetes prevalence between 1990 and 2008

The decomposition equation recognizes that the difference in diabetes prevalence between the two years, $p^{2008} - p^{1990}$, can be expressed as:

$$p^{2008} - p^{1990} = \sum_i (p_i^{2008} - p_i^{1990}) \left(\frac{c_i^{2008} + c_i^{1990}}{2} \right) + \sum_i (c_i^{2008} - c_i^{1990}) \left(\frac{p_i^{2008} + p_i^{1990}}{2} \right) \tag{1}$$

The first term on the right hand side is the contribution of differences in the age-specific levels of diabetes prevalence between the two years while the second term is the contribution of differences in the age-structure between the two years. Therefore, the percentage of the growth in diabetes between the two years attributable to changes in age-structure is calculated as:

$$\Delta_1 = \frac{\sum_i (c_i^{2008} - c_i^{1990}) \left(\frac{p_i^{2008} + p_i^{1990}}{2} \right)}{p^{2008} - p^{1990}} \tag{2}$$

Similarly, the percentage of the growth in diabetes between the two years attributable to changes in the age-specific levels of diabetes prevalence is:

$$\Delta_2 = \frac{\sum_i (p_i^{2008} - p_i^{1990}) \left(\frac{c_i^{2008} + c_i^{1990}}{2} \right)}{p^{2008} - p^{1990}} \tag{3}$$

Note that population growth would not affect the change in diabetes prevalence since equation (1) is purely based on proportionate measures (differences in growth rates across age groups is captured by the proportionate age distribution terms).

3 Further decomposition of the contribution of changes in the age-specific levels of diabetes prevalence

From the previous section, Δ_2 is the contribution of changes in the age-specific levels of diabetes prevalence to the change in overall diabetes prevalence between 1990 and 2008. To determine the relative importance of any age group j to this term, we began with the standard equation of Δ_2 developed above:

$$\Delta_2 = \frac{\sum_i (p_i^{2008} - p_i^{1990}) \left(\frac{c_i^{2008} + c_i^{1990}}{2} \right)}{p^{2008} - p^{1990}} \tag{4}$$

We then estimated a counterfactual version, Δ_2^j, where $p_i^{2008} - p_i^{1990} = 0$ when $i = j$:

$$\Delta_2^j = \frac{\sum_{i \neq j} (p_i^{2008} - p_i^{1990}) \left(\frac{c_i^{2008} + c_i^{1990}}{2} \right)}{p^{2008} - p^{1990}} \tag{5}$$

This counterfactual version represents the contribution of change in the age-specific levels of diabetes prevalence if the age-specific diabetes prevalence did not change for age group j. By comparing this value to the original value, Δ_2, we can measure the relative importance of age group j to Δ_2. Formally, the percent contribution of age group j to Δ_2 is:

$$\gamma_2^j = \frac{\Delta_2 - \Delta_2^j}{\Delta_2} \tag{6}$$

This was repeated for every age group to determine the percent contribution of each age group to Δ_2.
